# The Rumen Bacterial Community in Dairy Cows Is Correlated to Production Traits During Freshening Period

**DOI:** 10.3389/fmicb.2021.630605

**Published:** 2021-03-04

**Authors:** Shuai Huang, Shoukun Ji, Garret Suen, Feiran Wang, Shengli Li

**Affiliations:** ^1^State Key Laboratory of Animal Nutrition, Beijing Engineering Technology Research Center of Raw Milk Quality and Safety Control, College of Animal Science and Technology, China Agricultural University, Beijing, China; ^2^College of Animal Science and Technology, Hebei Agricultural University, Baoding, China; ^3^Department of Bacteriology, University of Wisconsin-Madison, Madison, WI, United States

**Keywords:** ruminal bacteria, fresh cows, dry matter intake, production traits, 16S rRNA sequencing

## Abstract

The rumen microbiome plays a vital role in providing nutrition to the host animal, thereby influencing ruminant production. Despite its importance, it is not fully understood how variation in the ruminal bacteria community composition influences dry matter intake (DMI), milk yield and ruminal fermentative parameters in dairy cows, especially during freshening period. Here, we hypothesized that during early lactation, high DMI cows having a different ruminal microbiota than low DMI cows, and that this difference persists over time. To test this, we enrolled 65 fresh and determinzed their DMI using an auto-feed intake recording system. Fourteen days after calving, the 10 animals with the lowest (LFI) and the 10 animals with the highest (HFI)-average DMI were selected for further analysis. Rumen fluid was collected from these two cohorts at 1 (Fresh1d) and 14 days (Fresh14d) after calving and their ruminal microbiota were assessed using 16S rRNA sequencing. Volatile fatty acid (VFA) concentrations were also quantified. Comparison of the ruminal microbiotas between Fresh1d and Fresh14d showed that Fresh14d cows had a significantly higher relative abundance of VFA—producing microbes (*P* < 0.05), such as *Prevotella_7* and *Succinivibrionaceae_UCG-001*. This was commensurate with the concentrations of acetate, propionate, butyrate, valerate and total VFAs, were also significantly (*P* < 0.05) increased in Fresh14d cows. We also found that the differences in the ruminal microbiota between LFI and HFI cows was limited, but DMI significantly altered (*P* < 0.05) the relative proportion of bacteria in the families *Coriobacteriaceae*, and *Succinivibrionaceae*. Furthermore, specific operational taxonomic units belonging to the *Anaeroplasma* was significantly (*P* < 0.05) correlated with DMI and milk yield. Taking together, our findings provide a framework for future studies of freshening period cow that seek to better understand the role of the ruminal microbiota during this critical period in the lactation cycle.

## Introduction

Dairy cows are important global contributors to agriculture as sources of milk and milk products. A critical stage in the dairy cow production lifecycle is the transition period, which occurs between lactation cycles and spans from 3 weeks before to 3 weeks after calving. During this period, cows undergo dramatic changes in host physiology and nutrient metabolism, which can result in health disorders, reduced dry matter intake (DMI), and lower milk yield. Previous works has documented the influence of diet on host metabolism and physiology during the transition period, but far less is known regarding the impact of the ruminal microbiome, which is a known driver of host production (Weimer, 2015). Importantly, ruminal microbes ferment plant polysaccharides into VFAs, including acetate, propionate, and butyrate, which serve as the major energy source for the cow (Reynolds et al., 1988; Flint et al., 2008). Recent studies reported the difference of rumen microbiota under the different feed intake of lactating dairy cows (Li et al., 2020) and yaks (Shi et al., 2020). However, there are no studies focused on the difference of rumen microbiota between low and high feed intake in dairy cows during the freshening period.

Recently, studies have shown that the rumen microbiota undergoes dramatic and distinct shifts from gestation to lactation. Lima et al. (2015) described these shifts in 115 Holstein dairy cows (67 multiparous and 48 primiparous) from 1 week before postpartum to 1 week after postpartum. Dynamic changes in the structure of the metabolically active rumen bacterial communities were found over the transition period (parturition ± 3 weeks), likely in response to the dramatic changes in physiology and nutritional factors like DMI and feed composition (Zhu et al., 2017). These findings were supported by another study of 10 primiparous Holstein dairy cows during the transition period, which also found distinct changes in the rumen bacterial composition in response to dietary changes (Zhu et al., 2018). In contrast, a study by Pitta et al. (2014) showed no difference in the dominant ruminal bacterial phyla, families and genera in both primiparous and multiparous cows 1–3 d post-calving and 4 weeks into lactation.

Given this paucity of data, it is clear that more work is required to better understand the influence of the ruminal microbiota during the transition period. In particular, a deeper understanding of the freshening period (2 weeks after parturition) is necessary, as ∼50% of all cows experience low DMI during this period, resulting in a state of negative energy balance (Ferguson, 2001). Increasing evidence showed that improving DMI of fresh cows can alleviate the negative energy balance and increased the downstream milk production of dairy cows (Roche et al., 2013).

Here, we hypothesized that the rumen microbiota of low DMI cows is significantly different from high DMI cows, and that this difference persists over time. To address this, we conducted a study to explore the dynamics of the ruminal microbial community during the freshening period in dairy cows. Specifically, we compared the ruminal microbiota of low DMI fresh cows to high DMI fresh cows in order to identify potential relationships between the ruminal bacteria and DMI. Understanding the differences between these groups will provide a framework for fresh cows and thereby achieve improved lactation efficiency while reducing the risk for the adverse outcomes that usually persist in low DMI fresh cows.

## Materials and Methods

### Animals Care and Management

Sixty-five fresh (2.40 ± 0.50 parity, body condition score 3.58 ± 0.12, body weight 612.13 ± 11.40 kg) Holstein dairy cows were selected after calving from a commercial dairy farm herd (Beijing, China). All cows were cohoused and kept in a free stall barn. No drugs or antibiotics were used 3 months prior to the study. All cows had *ad libitum* access to fresh water and were fed three times daily (07:30, 14:30, and 19:00) with a total mixed ration, as shown in Table 1.

**TABLE 1:** Composition and chemical components of the diet used in this study.

**Items^1^**	**Pre-partum**	**Fresh**
**Ingredients, kg**		
Oat grass	4.83	1.20
Alfalfa hay	–	3.00
Whole corn silage	8.62	11.20
Flaked corn	0.87	2.00
Corn pellets	0.87	1.18
Soybean meal	1.84	2.97
Soybean hull	0.50	2.00
Sprayed corn husk	1.40	0.18
DDGS	1.20	1.50
5% premix	0.75	0.65
Cottonseed	–	0.50
5% anion premix	0.13	–
Yeast culture XP	0.15	0.20
Molasses	–	0.50
**Contents,%**		
Dry matter as fed	53.39	49.32
Crude protein	14.23	16.56
Crude fat	2.04	3.40
ADF	20.37	20.58
NDF	40.84	34.63
NEL^2^(MCal/kg)	1.58	1.72
Ca	0.80	0.70
P	0.35	0.33

**^1^DDGS dried distillers grains with solubles, DM dry matter, NDF neutral detergent fiber, ADF acid detergent fiber, NFC non-fiber carbohydrates, Ca calcium, P phosphorus. ^2^NE_*L*_: Nutrients contents was measured value and NE_*L*_ was calculated value with model from NRC (2001).**

### Daily Milk Yield and Dry Matter Intake Data Collection

Individual feed intake was measured by a roughage intake control system (Insentec B.V., Marknesse, Netherlands). Cows were milked thrice daily (07:00, 14:00, and 22:00) by farm staff. Milk yield was recorded by a milking machine (2 × 48, BouMatic Company, Madison, WI, United States) for each cow.

### Grouping and Sampling Period

All cows were transferred to a new barn from 10 days before calving to allow the animals to acclimate to their new surroundings. The experimental period was from 1 d to 14 d after calving. During this period, the 10 cows with the lowest average DMI (LFI) and the 10 cows with highest average DMI (HFI) were selected from the 65 fresh cows. We note that a previous study found that characterizing the ruminal microbiota of 16 cows maintained on the same diet is sufficient to determine meaningful differences within their microbial communities (Jami and Mizrahi, 2012). Therefore, our selection of 20 disparate DMI cows from a cohort of 65 early lactation cows is likely sufficient to detect differences inthe their ruminal microbiotas as it relates to host phenotype. Once lactation began, the LFI and HFI cows were sampled at 1 (Fresh1d, *n* = 20) and 14 (Fresh14d, *n* = 20) days after calving.

### Blood Samples Collection and Measurement

Blood samples were collected from each cow via tail vein before morning feeding. Samples were centrifuged at 3,000 × g for 10 min to obtain serum and stored at −20°C untile subsequent analysis of glucose, non-esterified fatty acid (NEFA), and β-hydroxybutyrate (BHBA). Serum samples were analyzed for NEFA and BHBA using a colorimetric kit (Nanjing Jiancheng, Jiangsu, China), and glucose by a GF-D200 automatic biochemical analyzer (Caihong, Shandong, China).

### Rumen Fluid Collection and Processing

Rumen fluid samples were collected from each cow using an oral gastric tube (Ancitech, Winnipeg, MB, Canada) prior to morning feeding (07:00). The sampling device was cleaned thoroughly with fresh warm water after each sampling to avoid cow-to-cow contamination and the first 200 mL of collected rumen fluid was discarded to avoid saliva contamination. Subsequent rumen fluid was collected and filtered through four layers of cheesecloth. Samples were placed into sterile 50 mL plastic tubes on wet ice and immediately transported back to the farm office and frozen at −80°C until DNA extraction was performed.

An additional 30 mL of rumen fluid was transferred into a centrifuge tube and stored at −20°C until VFA analysis. VFA determination was conducted as follows. Rumen fluid was centrifuged at 8, 000 × g at 4°C for 15 min to obtain the supernatant, which was then quantified using gas chromatography as described by Erwin et al. (1961).

### Genomic DNA Extraction, Amplification, and Sequencing

Total genomic DNA was extracted from 1 mL rumen fluid samples using an OMEGA DNA kit (Omega Bio-Tek, Norcross, GA, United States) according to the manufacturer’s specifications. The quality of DNA was confirmed by 1% agarose gel electrophoresis. The amplicon library preparation was performed by PCR amplification of the V3-V4 region of the 16S rRNA gene using the primers 338F (5′-ACTCCTACGGGAGGCAGCAG-3′) and 806R (5′-GGACTACNNGGGTATCTAAT-3′) including NEBNext adapters sequences, indices and Taq DNA Polymerase as well as AMPure XP Beads (New England Biolabs Inc., Ipswich, MA, United States) (Ren et al., 2017). PCR conditions are as follows: 5 min of denaturation at 95°C, followed by 28 cycles of 45 s for denaturation at 95°C, 50 s for annealing at 55°C and 45 s for elongation at 72°C with a final extension at 72°C for 10 min. PCRs were performed in triplicate 25 μL mixture containing 12.5 μL KAPA 2G Robust Hot Start Ready Mix (Kapa Biosystems, Wilmington, MA, United States), 1 μL of each primer (5 μM), 5 μL template DNA (6 ng/uL) and 5.5 uL ddH_2_O. The amplified PCR products were purified using an Agencourt AMPure XP Kit (Beckman Coulter Genomics, Indianapolis, IN, United States), and quantified using PCR (ABI 9700, Thermo Fisher Scientific, Waltham, MA, United States). Purified PCR products were pooled in equimolar amounts and sequenced on an Illumina MiSeq (Illumina, San Diego, CA, United States) (Caporaso et al., 2012) using a 2 × 250 bp sequencing kit.

### Quality Control and Sequencing Data Analysis

Low quality (score ≤ 20) short reads (<200 bp) and reads containing ambiguous bases or unmatched to primer sequences and barcode tags were filtered out from dataset using QIIME 1.8 (Caporaso et al., 2010). The resulting reads were merged using PEAR 0.9.6 (Zhang et al., 2014) and demultiplexed using FLASH 1.20 (Magoc and Salzberg, 2011). Reads with merged length less than 230 bp and chimeric sequences were removed by UCHIME (UCHIME Algorithm) (Edgar et al., 2011). In order to reduce the error caused by the different sequencing depths of the samples, all samples were subsampled to equal size of 23,902 sequences for downstream alpha and beta diversity analysis. To ensure the comparability of the species diversity between the samples, standardized OTU documents were used to analyze the species and diversity indexes.

The remaining sequences were clustered into operational taxonomic units (OTUs) at a 97% similarity using the Ribosomal Database Project classifier (Cole et al., 2009) with a confidence threshold of 0.70 and compared against the SILVA 128 database (Release September 29, 2016) (Quast et al., 2013). All were removed using UCLUST (Edgar, 2010) to generate a representative OTU table.

The OTU level alpha diversity of bacterial communities was determined using Shannon and Chao1 indices and calculated using procedures within QIIME 1.8 and visualized using the “ggplot2” package in R (version 3.6.1) (Wickham, 2009). The non-metric multidimensional scaling (NMDS) ordination was performed on Bray-Curtis dissimilarity distances calculated in R. Analysis of similarities (ANOSIM) (999 permutations) using Bray-Curtis distances were performed to compare the similarity of microbial community among the observed microbial profiles based on different groups and sample time using the “vegan” package in R (Oksanen et al., 2015).

### Sequence and Statistics Analysis

Data on DMI, milk yield, rumen fermentation parameters and serum biochemical parameters were analyzed using the linear mixed models procedure of SAS 9.4 (Cary, North Carolina, United States). Alpha-diversity indices, the significance of the pairwise comparison between LFI and HFI groups and between Fresh1d and Fresh14d groups were analyzed using the Wilcoxon rank test using the ‘‘dplyr’’ package^1^ (author, H. Wickham, R. François, L. Henry, K. Müller; published date, 2018; version, 0.7.6) in R. Spearman’s rank correlation was used to identify the relationship between the relative abundance of OTUs and production traits of LFI and HFI cows using the ‘‘Psych’’ package^2^ (author, W Revelle; published date, 2016; version, 1.6.9) and visualized using the ‘‘corrplot’’ package^3^ (author, Taiyun Wei; published date, 2017; version, 0.84) in R. All *P*-value was corrected using a false discovery rate of 0.05 as described by Benjamini and Hochberg (1995) and false discovery rate corrected *P* < 0.05 were considered significant.

## Results

### Measurement of Production Traits, Rumen Fermentative Parameters, and Blood Metabolites in Fresh Cows

DMI and milk yield for all cows across the entire trial period are shown in Table 1. The levels of acetate, propionate, butyrate, valerate and total VFAs were significantly (*P* < 0.05) higher in Fresh14d compared to Fresh1d, whereas the acetate:propionate (AP) ratio and serum glucose was significantly (*P* < 0.05) lower in Fresh14d groups. We found that DMI increased and significantly differed (*P* < 0.05) between LFI and HFI groups (Table 2). No significant differences (*P* > 0.05) in milk yield, DMI/milk yield, acetate, propionate, butyrate, valerate, isovalerate, total VFAs, AP, NEFA, BHBA and glucose were observed between LFI and HFI groups.

**TABLE 2:** DMI, milk yield, rumen fermentative parameters and blood metabolites.

**Item^1^**	**Freshening period**		**SEM**	***P-*value**	**DMI**		**SEM**	***P-*value**
	**Fresh1d**	**Fresh14d**			**LFI**	**HFI**		
	**(*n* = 20)**	**(*n* = 20)**			**(*n* = 10)**	**(*n* = 10)**		
DMI, kg	6.43	15.36	0.60	<0.001	16.52	19.57	0.59	0.023
Milk yield, kg	20.47	36.41	1.45	<0.001	34.64	36.07	1.00	0.382
DMI/milk yield	0.74	0.50	0.05	<0.001	0.53	0.61	0.03	0.586
**Fermentation**								
Acetate, mmol/L	45.18	61.31	4.18	0.015	61.50	61.13	2.96	0.668
Propionate, mmol/L	10.96	20.22	1.63	0.002	20.74	19.64	1.75	0.781
Butyrate, mmol/L	5.77	10.25	0.75	<0.001	10.34	10.14	0.64	0.884
Valerate, mmol/L	0.53	1.01	0.06	<0.001	1.10	0.90	0.08	0.225
Isovalerate, mmol/L	2.10	2.05	0.12	0.779	2.18	1.92	0.12	0.269
TVFAs, mmol/L	63.14	94.20	6.89	0.005	93.86	94.74	4.95	0.936
AP	5.32	3.26	0.32	<0.001	3.24	3.29	0.16	0.883
**Metabolites**								
NEFA, mmol/L	0.20	0.21	0.002	0.058	0.21	0.20	0.005	0.170
BHBA, mmol/L	1.26	1.28	0.03	0.602	1.30	1.27	0.04	0.765
Glucose, mmol/L	3.65	3.11	0.12	0.025	3.14	3.08	0.12	0.815

*^1^TVFAs, total of VFAs; AP, the acetate-to-propionate raito; NEFA, non-esterified fatty acid; BHBA, β-hydroxybutyrate.*

### Sequencing Metrics for the Ruminal Microbiota of Fresh Cows

A total of 1,087,457 raw sequences were generated with an average of 27,186 ± 745 (mean ± *SD*) per sample, respectively. An average of 1,532 ± 258 OTUs across all samples was identified at 97% sequence similarity. Rarefaction curves showed a smaller number of new OTU identification as the number of sequences per sample increased (Supplementary Figure S1), implying the adequate sampling depth for covering the rumen bacterial composition that we tested. Good’s coverage for the fresh cow samples was determined with a mean value of 0.982 across all 40 samples, indicating sufficient sequence coverage for all samples. The mean Shannon’s diversity and Chao1’s richness for all fresh cow samples was 8.38 ± 0.64 and 1963.94 ± 285.88, respectively.

**FIGURE 1:**
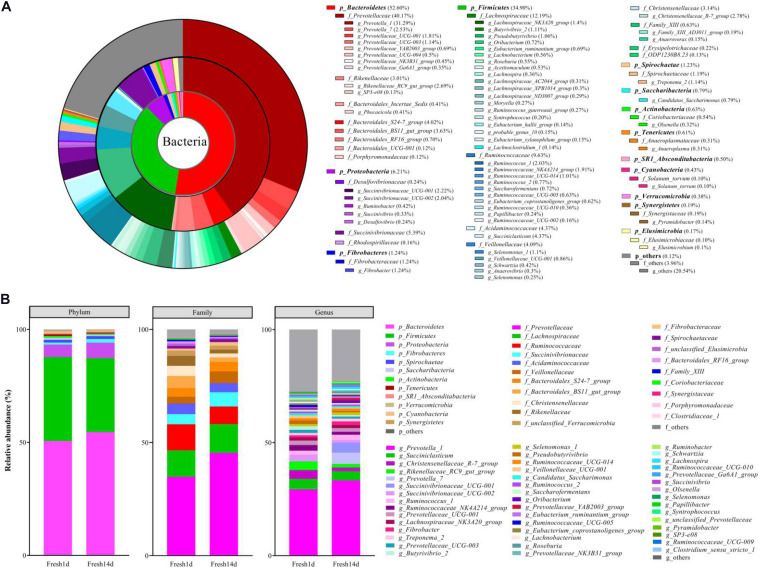
The ruminal bacterial composition of fresh cows. **(A)** Relative distribution of the most dominant bacterial phyla, family and genera (relative abundance >0.1% for all samples) for fresh cow samples. The pie chart inner ring represents the genus level, the middle ring represents the family level and the outer ring represents the phylum level. Different shades of color represent different bacteria. Numbers in brackets denote the average relative abundance of the bacteria across 40 samples. **(B)** Stacked bar graphs of the average relative abundances of phyla, family and genus (relative abundance >0.1% at least one sample) for fresh cow on d1 and d14 after calving.

The most highly abundant phyla for all fresh cow samples included the *Bacteroidetes* (52.60%), *Firmicutes* (34.90%), *Proteobacteria* (6.21%), with less contributions from the *Fibrobacteres* (1.24%) and *Spirochaetes* (1.23%) (Figures 1A,B). Within these phyla, the most abundant families included the *Prevotellaceae* (40.17%), *Lachnospiraceae* (12.19%), *Ruminococcaceae* (9.63%) and *Succinivibrionaceae* (5.39%, Figures 1A,B). At the genus level, 8 genera had >2% relative abundance: *Prevotella_1* (31.29%), *Succiniclasticum* (4.37%), *Christensenellaceae_R-7_group* (2.78%), *Rikenellaceae_RC9_gut_group* (2.69%), *Prevotella_7* (2.53%), *Succinivibrionaceae_UCG-001* (2.22%), *Succinivibrionaceae_UCG-002* (2.04%), and *Ruminococcus_1* (2.03%, Figures 1A,B).

### Defining a Core Microbiota for Fresh Cows and DMI Cows

**FIGURE 2:**
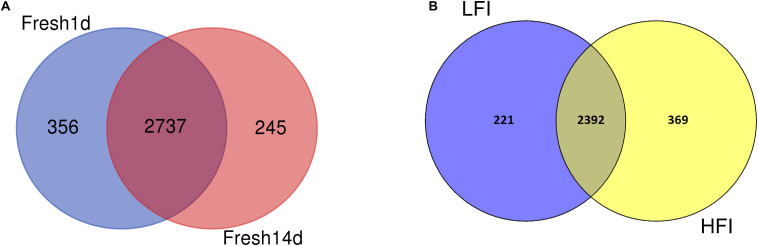
**(A)** Venn diagram plot of fresh cow samples. The core community for all fresh cow is defined as those OTUs present in all fresh animals cows for all sampling time. **(B)** Venn diagram plot of LFI and HFI cow samples. The core community for LFI and HFI cows is defined as those OTUs present in LFI and HFI cow at 14d after calving.

We then sought to determine the core microbiota across all fresh cows in our study and found 2,737 OTUs shared among all fresh cow samples (Figure 2A). These included bacterial families with >1% total relative abundance: *Prevotellaceae* (26.39%), *Lachnospiraceae* (6.23%), *Ruminococcaceae* (4.26%), *Acidaminococcaceae* (4.19%), *Bacteroidales_S24-7_group* (3.01%), *Veillonellaceae* (2.61%), *Christensenellaceae* (1.85%), and *Fibrobacteraceae* (1.05%, Supplementary Table S1). The shared genera among all samples >1% of the total relative abundance were the *Prevotella_1* (23.39%), *Succiniclasticum* (4.19%), *unclassified_Bacteroidales_S24-7_group* (3.01%), *Christensenellaceae_R-7_group* (1.85%), *Ruminocroccaceae_ NK4A214_group* (1.27%), *Lachnospiraceae_NK3A20_group* (1.11%), *Selenomonas_1* (1.06%), and *Fibrobacter* (1.05%).

We then determined the core set of OTUs shared between the LFI and HFI cows and found 2,392 OTUs shared across LFI and HFI samples (Figure 2B). More unique OTUs were found in the HFI group, relative to the LFI group. The LFI and HFI groups shared 80% of the total number of identified OTUs. Of the 2,392 OTUs shared across both DMI groups, most of them belonged to the genera *Prevotella_1* (33.26%), *unclassified_c_WCHB1-41* (21.80%), *Prevotella_7* (4.96%), *Succinivibrionaceae_UCG-001* (4.45%), *unclassified_Bacteroidales_S24-7_group* (4.20%), *Succiniclasticum* (4.05%), *unclassified_Lachnospiraceae* (2.69%), and *Ruminococcus_1* (2.32%, Supplementary Table S1).

### The Ruminal Bacterial Community in Fresh Cows Differs Between Days 1 and 14 After Calving

**FIGURE 3:**
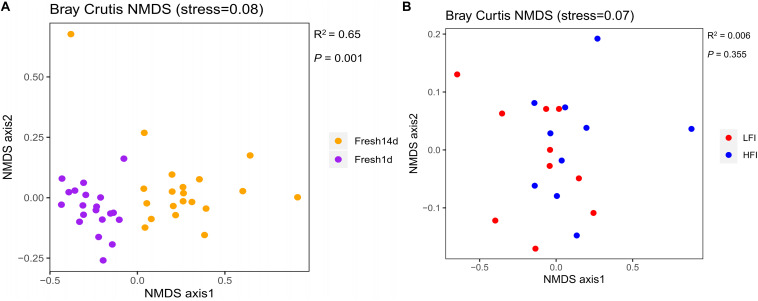
**(A)** NMDS plots of the bacterial communities of fresh cows samples on 1 d (*n* = 20) and 14 d (*n* = 20) after calving. Fresh1d: fresh cows on 1d after calving; Fresh14d: fresh cows on 14 d after calving. **(B)** NMDS plots of the ruminal bacterial communities of LFI (*n* = 10) and HFI (*n* = 10) cows based on Bray-Curtis distance. LFI, low feed intake fresh cow group; HFI, high feed intake fresh cow group.

To determine if differences exist between the ruminal microbiota of fresh cows at Fresh1d and Fresh14d, we performed a Bray-Curtis dissimilarity analysis and visualized this using an NMDS plot as shown in Figure 3A. We found that the ruminal microbiota differed between both groups upon visual inspection. We then analyzed these data in greater detail using ANOSIM and confirmed that these two groups were statistically different (*R*^2^ = 0.65, *P* = 0.001). Moreover, we found that the ruminal microbiota of the Fresh14d cows had a significantly (*P* < 0.001) lower number of OTUs, relative to Fresh1d cows, which was further supported by significant differences in the Chao1 richness and Shannon diversity index (*P* < 0.001) values for the two groups (Supplementary Figure S2A).

At the phylum level, the relative abundance of phyla *Bacteroidetes*, *Firmicutes* and *Proteobacteria* showed no significant (*P* > 0.05) difference between Fresh1d and Fresh14d. In contrast, the phyla *Actinobacteria* and *Tenericutes* were significantly (*P* < 0.05) decreased (Table 3). At the family level, the predominant family *Ruminococcaceae*, *Bacteroidales_BS11_gut_group*, *Christensenellaceae* and *Rikenellaceae* were significantly (*P* < 0.05) decreased from Fresh1d to Fresh14 (Table 3). The relative abundance of the families *Prevotellaceae* and *Veillonellaceae* were significantly (*P* < 0.05) higher in Fresh14d compared to Fresh1d (Table 3). At the genus level, the relative abundance of some genera changed more than 10-fold, including *Prevotella_7* (increasing 83.33-fold, *P* < 0.001), *Erysipelotrichaceae_UCG-002* (increasing 3827.75-fold, *P* = 0.001), and *Succinivibrionaceae_UCG-001* (increasing 42,380.95-fold, *P* < 0.001, Table 4).

**TABLE 3:** Significantly different phyla and families (relative abundance > 0.1%) within the rumen microbiota by lactation period and DMI as determined by the Wilcoxon test.

**Phylum/Family**	**Lactation**		**SEM**	***P-*value**	**DMI**		**SEM**	***P-*value**
	**Fresh1**	**Fresh14d**			**LFI**	**HFI**		
***Actinobacteria***	0.74	0.51	0.06	0.040	0.39	0.63	0.07	0.089
*Coriobacteriaceae*	0.66	0.43	0.05	0.011	0.32	0.54	0.06	0.045
***Bacteroidetes***	50.65	54.56	1.17	0.210	52.04	57.07	1.68	0.143
*Bacteroidales_BS11_gut_group*	5.30	1.95	0.42	<0.001	1.57	2.34	0.27	0.280
*Bacteroidales_Incertae_Sedis*	0.55	0.27	0.03	0.002	0.23	0.30	0.03	0.315
*Bacteroidales_RF16_group*	1.00	0.40	0.08	0.006	0.33	0.47	0.09	0.406
*Bacteroidales_UCG-001*	0.17	0.08	0.02	0.035	0.04	0.11	0.02	0.256
*Marinilabiaceae*	0.12	4.18E-03	0.02	0.002	2.09E-03	6.28E-03	1.49E-03	0.087
*Porphyromonadaceae*	0.21	0.02	0.04	0.002	7.53E-03	0.04	0.02	0.516
*Prevotellaceae*	34.89	45.45	1.70	0.002	44.07	46.82	2.07	0.315
*Rikenellaceae*	4.35	1.68	0.32	<0.001	1.27	2.09	0.29	0.315
***Firmicutes***	37.11	32.69	1.47	0.054	34.16	31.22	1.40	0.481
*Christensenellaceae*	4.53	1.76	0.32	0.002	1.30	2.22	0.35	0.063
*Clostridiales_vadinBB60_group*	0.11	0.03	0.01	0.010	0.02	0.04	8.91E-03	0.092
*Family_XIII*	0.80	0.46	0.04	0.002	0.46	0.46	0.03	0.791
*ODP1230B8.23*	0.17	0.08	0.03	0.011	0.02	0.13	0.05	0.140
*Ruminococcaceae*	11.49	7.77	0.55	0.007	7.96	7.58	0.53	0.796
*Veillonellaceae*	3.03	5.16	0.34	0.003	5.46	4.86	0.45	0.529
***Proteobacteria***	5.53	6.90	1.17	0.840	9.36	4.43	1.23	0.043
*Desulfovibrionaceae*	0.34	0.14	0.03	<0.001	0.13	0.15	0.02	0.940
*Succinivibrionaceae*	4.49	6.30	0.73	0.610	8.89	3.71	1.25	0.043
***Synergistetes***	0.18	0.19	0.08	0.005	0.10	0.28	0.09	0.186
*Synergistaceae*	0.18	0.19	0.04	0.060	0.10	0.28	0.09	0.186
***Tenericutes***	0.75	0.47	0.06	0.005	0.44	0.50	0.06	0.880
*Anaeroplasmataceae*	0.39	0.24	0.03	0.049	0.19	0.28	0.04	0.344

**TABLE 4:** Significantly different genera (relative abundance > 0.1%) within the rumen microbiota by lactation period and DMI as determined by the Wilcoxon test.

**Genera**	**Lactation**		**SEM**	***P-*value**	**DMI**		**SEM**	***P-*value**
	**Fresh1d**	**Fresh14d**			**LFI**	**HFI**		
*Phocaeicola*	0.55	0.27	0.03	<0.001	0.23	0.30	0.03	0.315
*Prevotella_7*	0.06	5.00	1.02	<0.001	7.80	2.19	1.90	0.344
*Prevotellaceae_Ga6A1_group*	0.22	0.49	0.04	0.003	0.41	0.58	0.06	0.290
*Prevotellaceae_UCG-003*	1.37	0.91	0.09	0.017	0.70	1.13	0.12	0.112
*Prevotellaceae_YAB2003_group*	0.32	1.07	0.10	<0.001	1.13	1.00	0.15	0.912
*Rikenellaceae_RC9_gut_group*	3.84	1.54	0.28	<0.001	1.19	1.88	0.24	0.315
*SP3-e08*	0.20	0.06	0.03	<0.001	0.01	0.11	0.05	0.494
*Anaerotruncus*	0.14	0.04	0.01	<0.001	0.05	0.03	0.01	1.000
*Anaerovorax*	0.20	0.09	0.01	<0.001	0.10	0.09	0.01	0.677
*Butyrivibrio_2*	1.57	0.66	0.10	<0.001	0.65	0.68	0.06	0.971
*Christensenellaceae_R-7_group*	3.90	1.66	0.28	<0.001	1.20	2.11	0.34	0.070
*Coprococcus_2*	0.02	0.06	0.01	0.003	0.07	0.06	0.01	0.426
*Erysipelotrichaceae_UCG-002*	2.09E-04	0.08	0.02	0.001	0.14	0.01	0.03	0.038
*Eubacterium_coprostanoligenes_group*	0.79	0.45	0.05	0.003	0.42	0.48	0.03	0.406
*Eubacterium_hallii_group*	0.16	0.12	0.01	0.018	0.09	0.14	0.02	0.173
*Eubacterium_ruminantium_group*	0.47	0.91	0.07	0.008	1.01	0.81	0.11	0.529
*Eubacterium_uniforme*	6.28E-04	0.10	0.02	<0.001	0.13	0.08	0.03	0.344
*Lachnoclostridium_1*	0.11	0.17	0.01	0.006	0.18	0.15	0.01	0.344
*Lachnoclostridium_12*	0.03	0.09	0.01	0.017	0.10	0.07	0.02	0.384
*Lachnospira*	0.05	0.67	0.09	<0.001	0.92	0.41	0.16	0.075
*Lachnospiraceae_ND3007_group*	0.39	0.18	0.04	0.001	0.21	0.16	0.05	0.850
*Lachnospiraceae_NK4A136_group*	0.13	0.07	0.01	0.013	0.08	0.06	0.01	0.449
*Lachnospiraceae_XPB1014_group*	0.47	0.14	0.03	<0.001	0.10	0.17	0.03	0.256
*Lactobacillus*	0.01	0.07	0.01	0.002	0.10	0.04	0.03	0.677
*Megasphaera*	0.01	0.11	0.02	<0.001	0.14	0.08	0.03	0.363
*Moryella*	0.31	0.22	0.02	0.010	0.22	0.23	0.02	0.970
*Oribacterium*	0.32	1.13	0.14	<0.001	1.42	0.84	0.25	0.529
*Papillibacter*	0.42	0.06	0.05	<0.001	0.05	0.07	0.01	0.384
*probable_genus_10*	0.18	0.11	0.01	0.039	0.12	0.11	0.02	0.850
*Pseudobutyrivibrio*	1.59	0.53	0.14	<0.001	0.46	0.59	0.06	0.353
*Ruminococcaceae_NK4A214_group*	2.51	1.30	0.14	<0.001	1.21	1.39	0.12	0.481
*Ruminococcaceae_UCG-002*	0.21	0.11	0.02	0.013	0.11	0.12	0.02	0.496
*Ruminococcaceae_UCG-005*	0.95	0.31	0.10	0.002	0.30	0.32	0.07	0.393
*Ruminococcaceae_UCG-010*	0.59	0.13	0.06	<0.001	0.11	0.16	0.03	0.054
*Schwartzia*	0.32	0.52	0.04	0.004	0.53	0.51	0.04	0.623
*Selenomonas*	4.81E-03	0.50	0.07	<0.001	0.65	0.35	0.13	0.315
*Selenomonas_3*	0.00	0.10	0.02	<0.001	0.15	0.07	0.03	0.103
*Veillonellaceae_UCG-001*	1.24	0.47	0.09	<0.001	0.44	0.50	0.05	0.473
*Desulfovibrio*	0.33	0.14	0.03	<0.001	0.13	0.15	0.02	0.910
*Ruminobacter*	0.68	0.17	0.09	<0.001	0.18	0.15	0.06	0.520
*Succinivibrionaceae_UCG-001*	1.05E-03	4.45	0.71	<0.001	6.48	2.42	1.24	0.162
*Succinivibrionaceae_UCG-002*	2.92	1.16	0.36	0.005	1.59	0.72	0.40	0.472
*Candidatus_Saccharimonas*	0.98	0.60	0.06	0.005	0.57	0.63	0.05	0.529
*Pyramidobacter*	0.13	0.15	0.03	0.018	0.09	0.21	0.06	0.186
*Anaeroplasma*	0.39	0.24	0.03	0.036	0.19	0.28	0.04	0.344

### Differences in the Ruminal Bacterial Community Between LFI and HFI Cows Is Limited

Considering the importance of DMI for the fresh group, we compared LFI and HFI cows from the fresh cow group at 14d to determine if differences exist between these cows. First, we performed a Bray-Curtis dissimilarity analysis of the microbiota for LFI and HFI cows and visualized this using an NMDS plot as shown in Figure 3B. We found that the ruminal microbiota was similar as both groups did not show a clear separation, and this was also confirmed using ANOSIM analysis (*R*^2^ = 0.006, *P* = 0.355). In addition, we found that the ruminal microbiota from those two groups had no significant differences in the Shannon diversity index (*P* = 0.529), Chao1 richness (*P* = 0.684), and number of OTUs (*P* = 0.481) for the two groups (Supplementary Figure S2B).

We then quantified the difference between the community composition of the LFI and HFI groups using the Wilcoxon test on the relative abundances for all samples at the phylum and family level. We found that phyla *Proteobacteria* was significantly (*P* = 0.043) enriched in the LFI group relative to the HFI group (Table 3). At the family level, *Coriobacteriaceae* (phylum *Actinobacteria*), and *Succinivibrionaceae* (phylum *Proteobacteria*) were significantly (*P* < 0.05) different between groups (Table 3). A higher relative abundance of *Erysipelotrichaceae_UCG-002* in the LFI group, relative to the HFI group (Table 4). The relative abundance of *Christensenellaceae_R-7_group* and *Ruminococcaceae_UCG-010* were tended to higher (0.05 < *P* < 0.1) and the relative abundance of *Lachnospira* was tended to lower (0.05 < *P* < 0.1) in the HFI group relative to the LFI group (Table 4).

### Correlation of Ruminal Bacteria With Production and Rumen Fermentative Parameters in LFI and HFI Cows

**FIGURE 4:**
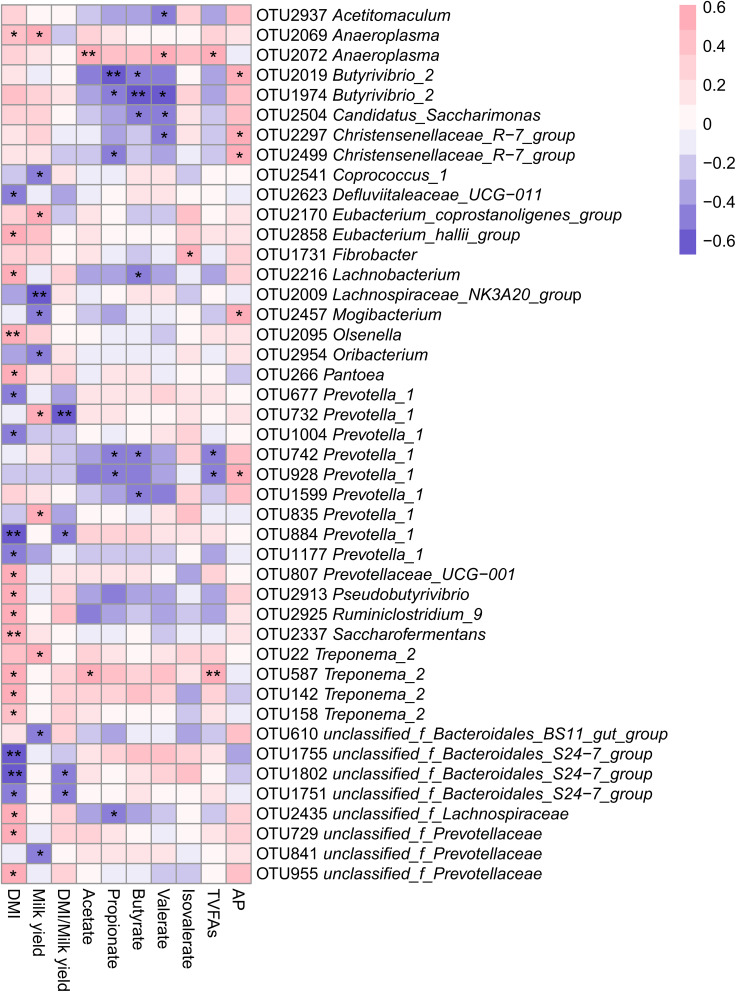
Heatmap of OTUs (relative abundance >0.01% in at all samples) significantly associated with production and rumen fermentative parameters in LFI and HFI cows, as determined by Spearman’s correlation analysis. *0.01 < *P* < 0.05, **0.001 < *P* < 0.01.

To explore the potential roles of ruminal bacteria on production and fermentation, we analyzed the relationship between DMI, milk yield, DMI/milk yield, VFAs (acetate, propionate, butyrate, valerate, isovalerate, total VFAs and AP) and the relative abundance of OTUs using Spearman’s rank correlations. All OTUs with relative abundances <0.01% of all samples were removed from this analysis. The relationship between OTUs and production and fermentation traits were visualized in a heatmap, as showed in Figure 4. We found a total of 24 OTUs that were significantly (*P* < 0.05) correlated with DMI; of them, 8 OTUs negatively correlated with DMI, 4 of which were in the genus *Prevotella_1* (*P* < 0.05) and 3 of which were in the family *Bacteroidales_S24-7_group* (*P* < 0.05). There were 15 OTUs positively correlated with DMI, of which 3 were in the *Treponema_2* (*P* < 0.05) and 2 were in the family *Prevotellaceae* (*P* < 0.05). There was one OTUs identified as belonging to the *Defluviitaleaceae_UCG-011* that was negatively (*P* < 0.05) associated with DMI. In addition, OTUs within the *Prevotellaceae_UCG-001*, *Lachnobacterium*, and *Olsenella* were significantly and positively (*P* < 0.05) correlated with DMI.

We also found 11 OTUs that were significantly correlated with milk yield; of them, 6 OTUs were significantly and negatively (*P* < 0.05) associated with milk yield, and belonged to the genera *Lachnospiraceae_NK3A20_group*, *Coprococcus_1*, *Oribacterium*, *Mogibacterium* and family *Bacteroidales_BS11_gut_group* and *Prevotellaceae*. There were 5 OTUs significantly and positively (*P* < 0.05) correlated with milk yield and belonged to the genera *Prevotella_1*, *Anaeroplasma*, *Treponema_2*, and *Eubacterium_coprostanoligenes_group*. Additionally, we identified a significant and negative correlation between DMI/milk yield and the relative abundance of OTUs within *Prevotella_1* and the family *Bacteroidales_S24-7_group* (*P* < 0.05).

For VFAs, we found that acetate concentration was postively correlated with the relative abundance of OTU2072 (*Anaeroplasma*, *r* = 0.578, *P* = 0.007) and OTU587 (*Treponema_2*, *r* = 0.550, *P* = 0.012). We also found that propionate concentration was negatively correlated with the relative abundance of four OTUs, including OTU2019 (*Butyrivibrio_2*, *r* = −0.654, *P* = 0.003), OTU742 (*Prevotella_1*, *r* = −0.469, *P* = 0.049), OTU928 (*Prevotella_1*, *r* = −0.523, *P* = 0.026), and OTU2499 (*Christensenellaceae_R-7_group*, *r* = −0.476, *P* = 0.046). The butyrate concentration was negatively and significantly (*P* < 0.05) correlated with OTUs within *Butyrivibrio_2*, *Candidatus_Saccharimonas*, and *Prevotella_1*. OTUs within *Anaeroplasma* and the family *Bacteroidales_S24-7_group* were significantly and positively (*P* < 0.05) correlated with valerate concentration, while OTUs within *Acetitomaculum*, *Butyrivibrio_2*, *Candidatus_Saccharimonas*, and *Christensenellaceae_R-7_group* were significantly and negatively (*P* < 0.05) correlated with valerate concentration. The isovalerate concentration was positively correlated with OTU1731 (Fibrobacter, *r* = 0.522, *P* = 0.018). The AP ratio was positively correlated with the relative abundances of OTU2019 (*Butyrivibrio_2*, *r* = 0.571, *P* = 0.013), OTU2297 (*Christensenellaceae_R-7_group*, r = 0.514, *P* = 0.029), OTU2499 (*Christensenellaceae_R-7_group*, *r* = 0.491, *P* = 0.038), OTU2457 (*Mogibacterium*, *r* = 0.502, *P* = 0.034) and OTU928 (*Prevotella_1*, *r* = 0.504, *P* = 0.033).

## Discussion

The objective of this study was to characterize the ruminal microbiota during the freshening period and to determine the impact of DMI in shaping its dynamics. We designed this experiment to follow the rumen microbiota within the first 14 days after calving in a group of fresh cows and to compare low and high DMI cows from this group. Given that low DMI in fresh cows is known to result in reduced lactation efficiency and increased risk for host metabolic syndromes, understanding the dynamics of the ruminal microbiota during this period may provide a framework for managing this critical transition period.

Consistent with the known changes in both host metabolism and the endocrine system across gestation and lactation, it is perhaps not surprising that we observed significant differences in the ruminal bacterial community and rumen fermentation index in fresh cows between days 1 and 14. This is also likely due to the significant differences in the diet fed to transition animals, which differs substantially from the beginning to the end of this period. Recently it was shown that lactation has a far greater impact in shaping the ruminal microbiota in dairy cows than host genetics (Bainbridge et al., 2016). Additionally, Pitta et al. (2014) found that the ruminal microbiota of lactation cows 1–3 days after calving was most similar to the ruminal microbiota of prepartum cows. Thus, the shifts in the ruminal bacterial community of fresh cows from 1 to 14 days observed here is likely due to the interaction of lactation and diet.

Compared to Fresh1d cows, the Fresh14d cows harbored a higher relative abundance of *Prevotellaceae*, *Veillonellaceae*, and bacteria within these families are known to degrade and ferment carbohydrates into VFAs (Zhang et al., 2018). We also found significantly higher relative abundances of *Succinivibrionaceae_UCG-001*, which was increased more than 4,000-fold in Fresh14d cows. This is in agreement with observations indicating that members of this genera utilize hydrogen to produce succinate, which can be converted to propionate (McCabe et al., 2015). This purported in propionate may contribute to the observed decrease in AP. Moreover, we found higher levels of acetate, propionate, valerate, total VFAs, and a lower AP ratio in the rumen from Fresh14d cows, relative to Fresh1d cows, which likely reflects a stronger fermentation capacity of the Fresh14d ruminal microbiota.

In addition to our findings on the temporal dynamics of the rumen microbiota in fresh cows, our study also considered the impact of DMI on the rumen microbiota of fresh cows. Here, we found that increased DMI was associated with lower relative abundances of *Erysipelotrichaceae_UCG-002*, within the family *Erysipelotrichaceae*, and higher relative abundances of *Ruminococcaceae_UCG-010*, within the family *Ruminococcaceae*. These findings are in accordance with a previous study of low, medium, and high feed intake cows during early lactation which found decreased numbers of *Erysipelotrichaceae* and increased numbers of *Ruminococcaceae* in high feed intake cows (Li et al., 2020). More recently, a study on the feed efficiency of dairy cows found that increased milk production was associated with higher relative abundances of bacteria in the *Bacteroidales*, *Lachnospiraceae*, *Ruminococcaceae*, and *Prevotella* (Shabat et al., 2016).

Given the effect of DMI on the rumen bacterial community, it is not surprising that specific bacterial species are strongly correlated with DMI. Here, we observed a strong correlation between DMI and bacteria in the families *Prevotellaceae*, *Ruminnococcaceae*, *unclassified_Bacteroidales_S11_gut_group*, and *Lachnospiraceae*. This is in accordance with other studies that also found a strong correlation between DMI and bacteria in the families *Prevotellaceae* and *Ruminnococcaceae* (Jami et al., 2014). Other work demonstrating the heritability of OTUs within the *Succinivibrionaceae*, *Megasphaera*, *Selenonmonas*, *Oscillospira*, and unclassified *BS11*, as it relates to DMI in beef cattle (Li et al., 2019), also support our findings. Related to this, our study also found that OTUs associated with high DMI fresh cows were the core microbiota in LFI and HFI cows. We note that many of these are consistent with previously reported OTUs found to be heritable in high DMI beef cattle, including bacteria in the *Prevotella* and *Lanchnospirraceae* (Sasson et al., 2017). In our study, we also found a strong and positive correlation between milk yield and OTUs within *Prevotella_1*, *Anaeroplasm* and *Treponema_2*. This is in accordance with other studies which also found that *Prevotella*, *unclassified_Bacteroidales*_*S24-7* and *Succinivibrionaceae* were strongly and positively correlated with milk yield in lactating dairy cows (Indugu et al., 2017). Given these findings future work should further investigate these bacteria as potential targets for improving DMI in fresh cows.

Considering the essential role of the rumen bacteria in fermenting plant material into VFAs (Kittelmann et al., 2013), which have a direct effect on milk production (Hurtaud et al., 1995; Brulc et al., 2009), documenting the rumen microbiota in early lactation may help in better understanding the impact of the rumen microbiota on production traits. Moreover, the rumen microbiota during the freshening period may serve as a predictor of future production and may allow for manipulation in order to improve long term milk production. The results presented here have identified a number of specific bacterial taxa associated with both low and high DMI in fresh cows over time, and many of these may serve as potential targets for mitigating the challenges associated with low DMI cows during the freshening period. However, future work using more functional approaches, such as metagenomics and metatranscriptomics, should be conducted to better understand the interaction between rumen microbiome and DMI in fresh cows.

## Conclusion

In summary, the results of this study provide novel evidence for an alteration of the microbiome in the rumen of fresh cows from 1 to 14 days after calving. We found that the ruminal microbiota and its associated fermentation patterns differed during this period and that the relative abundance of many VFA—producing microbes within the *Prevotellaceae*, *Lactobacillaceae*, and *Veillonellaceae* were dramatically increased in Fresh14d cows compared with Fresh1d cows. These findings indicate a potential stronger ability to ferment dietary substrates by the rumen microbiota of Fresh14d cows than that of Fresh1d cows. Additionally, we found limited differences between the ruminal microbiota of LFI and HFI groups, thereby reflecting the limited role of DMI on shaping the rumen microbiota during the freshening period. Furthermore, a strong relationship between the relative abundances of specific OTUs and host production traits suggests the possibility to predict downstream host production using the rumen microbiota. This could lead to approaches for manipulating the rumen microbiota to improve DMI and milk production in dairy cows during the transition period. Future studies should investigate the relationship between the rumen microbiota and DMI across different environments in an integrative manner that incorporates both host genetics and functional metagenomics in the rumen.

## Data Availability Statement

The datasets presented in this study can be found in online repositories. The names of the repository/repositories and accession number(s) can be found below: https://www.ncbi.nlm.nih.gov/, PRJNA599409.

## Ethics Statement

The animal experiments and study protocols described in this study were approved by the Institutional Animal Care and Use Committee of the College of Animal Science and Technology (Project number 31772628) at China Agricultural University, Beijing, China.

## Author Contributions

SJ, SL, and SH conceived and designed the study. SH and FW collected all samples used in this study. SH performed the data analysis and wrote the manuscript with contributions from GS. All authors read and approved the final manuscript.

## Conflict of Interest

The authors declare that the research was conducted in the absence of any commercial or financial relationships that could be construed as a potential conflict of interest.

## Footnotes

1 https://cran.r-project.org/package/dplyr

2 http://cran.r-project.org/web/packages/psych

3 https://cran.r-project.org/web/packages/corrplot/

## Abbreviations

ADF: Acid detergent fiber
AP: acetate-to-propionate ratio
DDGS: Dried distillers grains with soluble
DM: Dry matter
DMI: Dry matter intake
Fresh1d: 1 day after calving of fresh cows
Fresh14d: 14 days after calving of fresh cows
HFI: high average feed intake from the fresh cows
LFI: low average feed intake from the fresh cows
NDF: Neutral detergent fiber
NMDS: Non-Metric Multidimensional Scaling
OTUs: Operational Taxonomic Units
TVFAs: total of volatile fatty acids.
