# Supplementary material for: The Rumen Bacterial Community in Dairy Cows Is Correlated to Production Traits During Freshening Period
Source: Front Microbiol. 2021 Mar 4;12:630605. doi: 10.3389/fmicb.2021.630605 (PMC7969525; doi:10.3389/fmicb.2021.630605)
Supplement: Supplementary file 2 [file Table_2.DOCX]

#
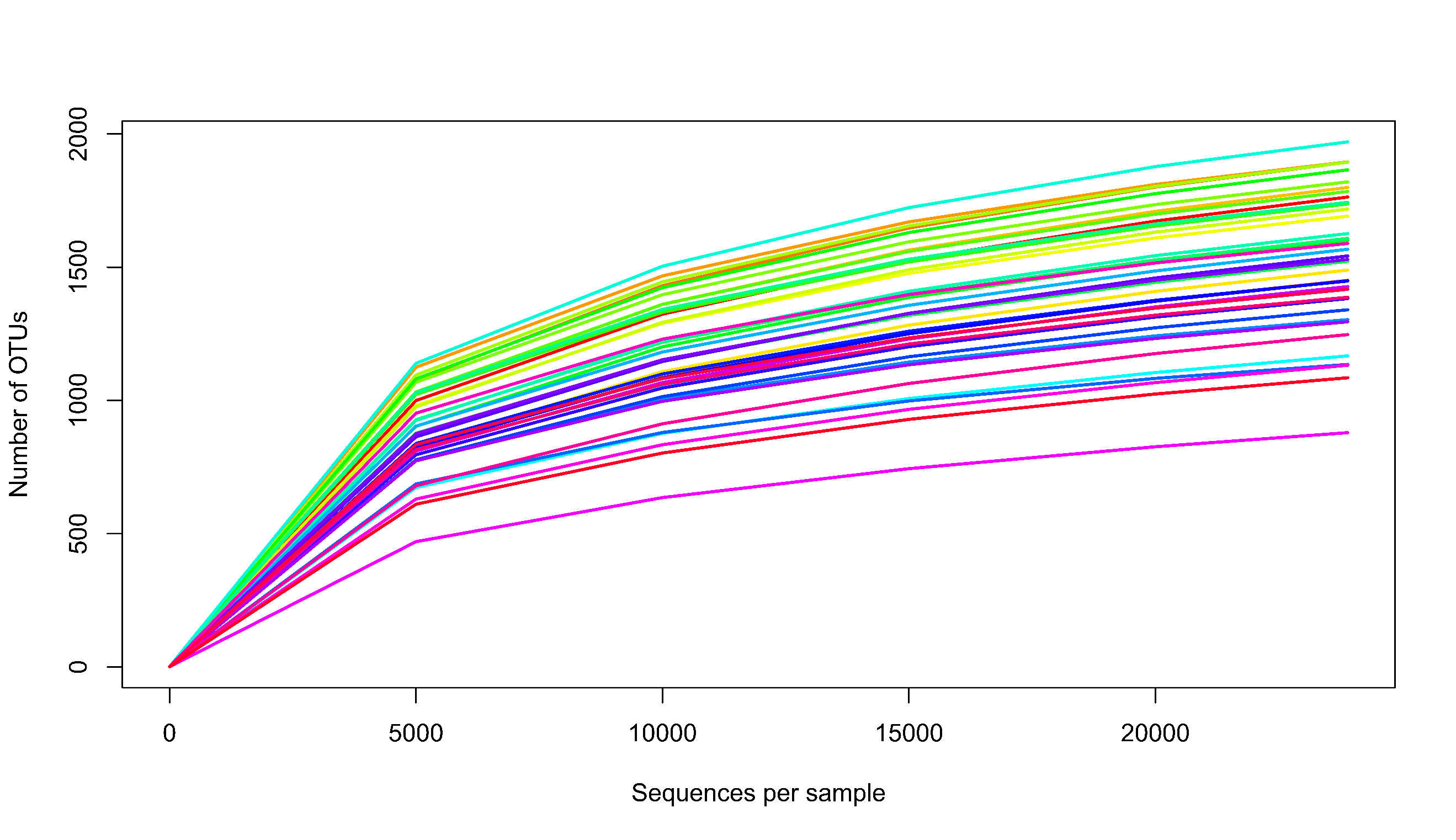
Figure S1 | Individual OTU rarefaction curves for each fresh cow rumen sample take. The smaller number of new OTUs increased as the sequence number increased, indicating that our sampling depth was adequate cover the rumen microbial composition we tested.

#
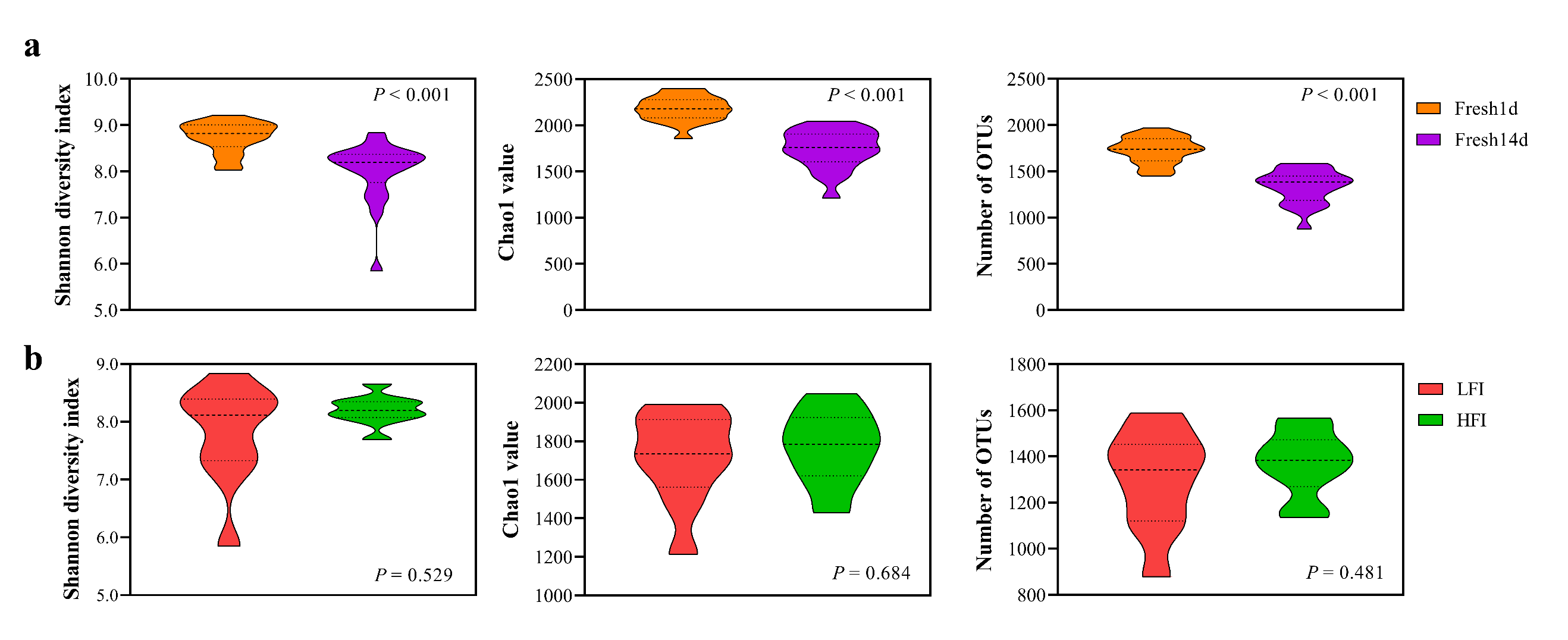
Figure S2 | Violin plots of number of OTUs, Chao1 richness and Shannon diversity index between Fresh1d and Fresh14d (a) and between LFI and HFI (b).
